# Readmission and associated clinical factors among individuals admitted with bipolar affective disorder at a psychiatry facility in Uganda

**DOI:** 10.1186/s12888-023-04960-0

**Published:** 2023-06-28

**Authors:** Joan Abaatyo, Mark Mohan Kaggwa, Alain Favina, Andrew T. Olagunju

**Affiliations:** 1 Department of Psychiatry, Faculty of Medicine, Mbarara University of Science and Technology, Mbarara, Uganda; 2 Department of Psychiatry and Behavioral Neurosciences, McMaster University, Hamilton, ON Canada; 3 Discipline of Psychiatry, University of Adelaide, Adelaide, SA 5000 Australia

**Keywords:** Bipolar affective disorder, Clinical symptoms, Mood symptoms, Mood lability, Readmission, Relapse

## Abstract

**Background:**

Bipolar affective disorder (BAD) is a common severe mental health condition with a relapsing course that may include periods of hospital re-admissions. With recurrent relapses and admissions, the course, prognosis, and patient’s overall quality of life can be affected negatively. This study aims to explore the rates and clinical factors associated with re-admission among individuals with BAD.

**Method:**

This study used data from a retrospective chart review of all records of patients with BAD admitted in 2018 and followed up their hospital records for four years till 2021 at a large psychiatric unit in Uganda. Cox regression analysis was used to determine the clinical characteristics associated with readmission among patients diagnosed with BAD.

**Results:**

A total of 206 patients living with BAD were admitted in 2018 and followed up for four years. The average number of months to readmission was 9.4 (standard deviation = 8.6). The incidence of readmission was 23.8% (*n* = 49/206). Of those readmitted during the study period, 46.9% (*n* = 23/49) and 28.6% (*n* = 14/49) individuals were readmitted twice and three times or more, respectively. The readmission rate in the first 12 months following discharge was 69.4% (*n* = 34/49) at first readmission, 78.3% (*n* = 18/23) at second readmission, and 87.5% (*n* = 12/14) at third or more times. For the next 12 months, the readmission rate was 22.5% (*n* = 11/49) for the first, 21.7% (*n* = 5/23) for the second, and 7.1% (*n* = 1/14) for more than two readmissions. Between 25 and 36 months, the readmission rate was 4.1% (*n* = 2/49) for the first readmission and 7.1% (*n* = 1/14) for the third or more times. Between 37 and 48 months, the readmission rate was 4.1% (*n* = 2/49) for those readmitted the first time. Patients who presented with poor appetite and undressed in public before admission were at increased risk of being readmitted with time. However, the following symptoms/clinical presentations, were protective against having a readmission with time, increased number of days with symptoms before admission, mood lability, and high energy levels.

**Conclusion:**

The incidence of readmission among individuals living with BAD is high, and readmission was associated with patients’ symptoms presentation on previous admission. Future studies looking at BAD using a prospective design, standardized scales, and robust explanatory model are warranted to understand causal factors for hospital re-admission and inform management strategies.

## Introduction

Bipolar affective disorder (BAD) is a severe, chronic mental illness with an estimated prevalence range of 1–5% worldwide [1, 2]. Despite the low prevalence, mortality is approximately three times greater, and life expectancy is reduced by 8–12 years in people with BAD compared to the general population [3, 4]. Furthermore, its considerable neuropsychiatric sequelae, medical comorbidities, and psychosocial dysfunction make BAD one of the leading causes of disability worldwide [5, 6]. Clinically, BAD is regarded as a recurrent or relapsing condition, characterized by periods of hypomania/mania and depression, with approximately one-third of patients relapsing into depression or mania within one year following an episode [7]; and potentially requiring readmission despite being on treatment [8]. Therefore, a comprehensive appreciation of the course of the illness and the factors associated with relapse is vital for optimum management and good outcomes [9].

The readmission rate of BAD is an important proxy indicator of the frequency of relapse, quality of care, prognosis, and a major focus of scrutiny for quality improvement for hospitals, health sector administrators, and policymakers [10]. Recurrent admission in individuals with BAD is often associated with functional impairment and a poor quality of life [11]. Living with BAD poses an enormous burden to the patients, their families or caregivers, and the society due to the high direct costs of treatment and indirect costs from reduced employment, productivity, and functioning [12–14]. Therefore, a better understanding of the factors associated with readmission and the length of community tenure can assist with the identification of high-risk patients for indicated preventive interventions. In this respect, various factors have been associated with re-admission in previous literature, including prior psychiatric hospitalizations, sleep deprivation, impaired functioning, psychological stressors, mental status instability, housing instability, and substance use problems [15, 16]. However, the clinical predictors like symptom profile and treatment-related factors associated with relapse and rehospitalization in patients with BAD following discharge remain understudied to a large extent in developing countries [17, 18]. Most studies assessing the clinical factors that are explanatory of readmission have been conducted in mixed-diagnosis samples outside Africa or all patients with a mental illness diagnosis [8, 19–21]. For example, one study in the USA found that symptoms consistent with mania decrease the likelihood of readmission, whereas those consistent with depression increase the likelihood [19].

Considering the gaps mentioned above and the significant burden of BAD on the affected individuals and the society, urgent research is needed to understand the rates of readmission and the predictors of time to readmission for patients with BAD in developing contexts, especially Africa. Using symptoms and other related clinical factors at admission is a cost-effective, easy to implement, and reliable way to predict individuals who may potentially relapse and require readmission [19]. In low-income settings such as Uganda, a good knowledge of factors associated with readmission (e.g., symptoms profile) could promote better recognition and characterization of at-risk patients and inform an efficient allocation of limited healthcare resources to mitigate readmission and manage patients in the community [22, 23]. In addition, such knowledge would further foster and inform community-based care of individuals with BAD [24]. Therefore, the present study examines the rates of readmissions, clinical factors associated with the readmission among individuals with BAD at a psychiatric unit in a tertiary referral hospital in Uganda. In this study, we postulated that identifiable clinical factors and symptom profile of BAD on admission would be associated with readmission.

## Methods

We conducted a retrospective cohort study. This study was based on a retrospective chart review of the inpatients with a diagnosis of BAD in 2018 at a psychiatric unit in a tertiary hospital in Uganda and followed for four years. The study was approved by the research ethics committee (Ethics number; MUST-2021-229). The psychiatry unit is the largest psychiatric facility in southwestern Uganda, with a mental health team (responsible for diagnosis, prescription and care) that consists of three psychiatrists and five psychiatry clinical officers during the study period. The facility on average, admits 14 individuals per week [25]. Details about the facility are well described in previous publications [26–29]. The unit provided clinical care and treatment to individuals with all categories of mental illnesses, including BAD, especially with referrals from the entire southwestern Uganda. The clinicians make the clinical diagnoses based on Diagnostic Statistical Manual, fifth edition (DSM-5) and/or International Classification of Diseases, tenth edition (ICD-10), but also document the patient clinical presentation as described by caregivers or informants.

### Inclusion and exclusion criteria

We included the records of individuals with a clinical diagnosis of BAD admitted in 2018 and followed up their records to gather relevant information on readmission for a period of four years. We excluded individuals who were readmitted within 30 days of discharge throughout the four years of follow-up because readmission was defined as a period of inpatient admission after 30 days from the last hospital admission. Most individuals admitted within 30 days, might still be experiencing symptoms from the same episode.

### Data collection

Information on the following variables were collected to answer the research questions in the current paper: (i) age, (ii) gender, (iii) year of admission, (iv) marital status, (v) level of education, (vi) occupation, (vii) treatment given, (viii) history of stressor, (ix) use of substances, (x) date of admission, (xi) presenting symptoms, (xii) duration of the presenting symptoms, (xiii) family history of mental illness, (xiv) length of hospital stay, (xv) time to readmission, and (xvi) administered psychotherapy during admission. Note: The individuals who are at the psychiatric facility, are given an identification number (in-patient number) at the first visit when they hospitalized; this number was used to track patients who were re-admitted over the defined study period. Data cleaning was done in Excel.

### Data analysis

Data were analyzed using STATA version 15.0. Categorical variables were presented with frequencies and percentages. Numerical variables were presented in terms of mean and standard error. To address the missing data for some of the variables examined, data analysis was done using complete-case analyses method [30, 31], and the frequency (*n*) for complete data for such item were shown. Chi-square tests were conducted to determine significant differences in sociodemographic characteristics of study participants with and without readmission during the study period. The distribution of clinical characteristics of individuals was compared across the different times of admission using Analysis of Variance (ANOVA). We defined readmission rate as the incidence proportion of readmission during the study period [3, 32]. Time to re-admission was calculated as the number of days from one admission to another. Kaplan-Meier curves were used to describe survival (time to readmission). Cox regression models were used to estimate hazard ratios and their 95% confidence intervals between readmission (dependent variable) and each clinical factor (independent variable) characteristic while controlling for social demographic characteristic [33]. We tested the proportional hazards assumption using the proportional hazard test in STATA (estat phtest, test). This suggested that no variables violated the assumption.

## Results

### Baseline characteristics of the study participants

Table 1 shows the baseline social demographic characteristics of the study participants. The mean age of individuals with BAD admitted in 2018 was 31.2 (*SE* = 0.7), with an almost equal number of males versus females (i.e., 49.5% vs. 50.5%). There were no significant differences in social demographic characteristics between readmitted patients and those who were never readmitted.

**Table 1:** Distribution of the social demographic characteristic of individuals with bipolar affective disorder with and without readmission an at baseline (2018)

Variable	All participants in 2018 n (%)	Participants with readmission		t/ χ^2^ (p-value)
		No n (%) 157 (76.2)	Yes n (%) 49 (23.8)	
**Age (mean, S.E)**	31.2 (0.9)	31.7 (1.0)	29.5 (1.6)	1.09 (0.277)
**Gender**				
Female	104 (50.5)	77 (49.0)	27 (55.1)	0.75 (0.386)
Male	102 (49.5)	80 (51.0)	22 (44.9)	
Level of education*				
None	37 (18.0)	28 (17.8)	9 (18.4)	7.32 (0.119)
Primary	32 (15.5)	29 (18.5)	3 (6.1)	
Secondary	50 (24.3)	34 (21.7)	16 (32.6)	
Tertiary	22 (10.7)	19 (12.1)	3 (6.1)	
Missing	65 (31.5)	47 (30.0)	18 (36.7)	
Marital status*				
Divorced	1 (0.5)	1 (0.6)	0 (0.0)	4.69 (0.455)
Married/cohabiting	78 (37.9)	57(36.3)	21 (42.9)	
Separated	14 (6.8)	9 (5.7)	5 (10.2)	
Single	85 (41.3)	65 (41.4)	20 (40.8)	
Widowed	3 (1.5)	3 (1.9)	0 (0.0)	
Missing	25 (12.1)	22 (14.0)	3 (6.1)	
Positive family history of mental illness*				
No	166 (80.6)	128 (81.5)	38 (77.5)	0.38 (0.539)
Yes	40 (19.4)	29 (18.5)	11 (22.5)	
Occupation*				
Unemployed	142 (68.9)	107 (68.1)	35 (17.4)	0.19 (0.911)
Employed	23 (11.2)	18 (11.5)	5 (10.2)	
Missing	41 (19.9)	32 (20.4)	9 (18.4)	

SE – Standard error; * - missing data from hospital records

### Clinical characteristics of study participants and readmissions during the years of follow up

At baseline in 2018, a total of 206 individuals were admitted with a diagnosis of BAD (T0). Of the 206, 49 (23.8%) individuals had their first readmission episode (R1) spread out over the different years of study from 2018 to 2021. Of the 49 that had their first readmission, 23 (46.9%) individuals had a second readmission (R2) and of the 23, 14 (60.9%) had more than two readmissions spread out over the entire study period (R3).

Note. The average number of months to readmission was 9.4 (standard deviation (*SD*) = 8.6).

During the duration of the study, it was observed that there was a significant statistical increase of participant having irritability in their clinical symptoms during subsequent re-admissions (χ^2^ = 7.86, p = 0.049). Psychotherapy was statistically offered more with subsequent readmissions (χ^2^ = 20.28, p < 0.001). However, there was a statistically significant lower proportion of participants with readmissions who presented with uncoordinated speech (χ^2^ = 12.24, *p* = 0.007), physical aggression (χ^2^ = 14.97, *p* = 0.002), and wandering away from home χ^2^ = 16.19, *p* = 0.001) with subsequent readmissions. For details, see Table 2.

**Table 2:** Clinical characteristics of study participants and readmissions across the follow up period

Variables	T0 (First admission in 2018 n (%) (n = 206)	R1 (Re-admitted) n (%) (n = 49)	R2 (Second re-admission) n (%) (n = 23)	R3 (Third and/or more re-admissions) n (%) (n = 14)	χ^2^ / F (p-value)
**Duration with symptoms before admission (in days) (mean, S.E)**	56 (14.8)	7 (1.2)	5 (10.9)	10 (3.1)	1.26 (0.288)
**Days of admission (mean, S.E)**	11 (1.0)	12 (4.1)	10 (1.8)	8 (2.2)	0.24 (0.870)
**Psychotherapy treatment given**					
No	146 (70.8)	25 (51.0)	7 (30.4)	6 (42.9)	**20.28 (< 0.001)**
Yes	60 (29.2)	24 (49.0)	16 (69.6)	8 (57.1)	
**SYMPTOMS AT PRESENTATION IN HOSPITAL**					
**Hallucinations**					
No	159 (77.2)	45 (91.8)	21 (91.3)	12 (85.7)	7.52 (0.057)
Yes	47 (22.8)	4 (8.16)	2 (8.7)	2 (14.3)	
**Delusions**					
No	173 (84.0)	44 (89.8)	20 (87.0)	12 (85.7)	1.13 (0.771)
Yes	33 (16.0)	5 (10.2)	5 (13.0)	2 (14.3)	
**Uncoordinated speech**					
No	143 (69.4)	44 (89.8)	19 (82.6)	13 (92.9)	**12.24 (0.007)**
Yes	63 (30.6)	5 (10.2)	4 (17.4)	4 (7.1)	
**Suspiciousness**					
No	194 (94.2)	47 (95.9)	23 (100)	14 (100)	2.40 (0.494)
Yes	12 (5.8)	2 (4.1)	-	-	
**Poor sleep**					
No	74 (35.9)	16 (32.6)	5 (21.7)	1 (7.1)	6.36 (0.095)
Yes	132 (64.1)	33 (67.4)	18 (78.3)	13 (92.9)	
**Physical aggression**					
No	88 (42.7)	22 (44.9)	19 (82.6)	9 (64.3)	**14.97 (0.002)**
Yes	118 (57.3)	27 (55.1)	4 (17.4)	4 (35.7)	
**Food refusal**					
No	194 (94.2)	47 (95.9)	20 (87.0)	12 (85.7)	3.64 (0.303)
Yes	12 (5.8)	2 (4.1)	3 (13.0)	2 (14.3)	
**Affective lability**					
No	179 (86.9)	43 (87.8)	18 (78.3)	13 (92.9)	1.93 (0.588)
Yes	27 (13.1)	6 (12.2)	5 (21.7)	1 (7.1)	
**Talking to self**					
No	189 (91.7)	45 (91.8)	21 (91.3)	13 (92.9)	0.03 (0.999)
Yes	17 (8.3)	4 (8.2)	2 (8.7)	1 (7.1)	
**Restlessness**					
No	129 (62.6)	27 (55.1)	11 (47.8)	9 (64.3)	2.61 (0.457)
Yes	77 (37.4)	22 (44.9)	12 (52.2)	5 (35.7)	
**Self-neglect**					
No	180 (87.38)	44 (89.8)	22 (95.7)	13 (92.9)	1.77 (0.622)
Yes	26 (12.6)	5 (10.2)	5 (10.2)	1 (7.1)	
**Destructiveness**					
No	171 (83.0)	39 (79.6)	22 (95.7)	13 (92.9)	3.98 (0.264)
Yes	35 (17.0)	10 (20.4)	1 (4.4)	1 (7.4)	
**Singing**					
No	188 (91.3)	46 (93.9)	20 (86.9)	13 (92.9)	1.01 (0.800)
Yes	18 (8.7)	3 (6.1)	3 (13.1)	1 (7.1)	
**Increased religiosity**					
No	192 (93.2)	46 (93.9)	22 (95.7)	14 (100)	1.19 (0.755)
Yes	14 (6.8)	3 (6.1)	1 (4.3)	-	
**High energy levels**					
No	150 (72.8)	34 (69.4)	18 (78.3)	11 (78.6)	0.87 (0.833)
Yes	56 (27.2)	15 (30.6)	5 (21.7)	3 (21.4)	
**Wandering away from home**					
No	132 (64.1)	43 (87.8)	19 (82.6)	13 (92.9)	**16.19 (0.001)**
Yes	74 (35.9)	6 (12.2)	4 (17.4)	1 (7.1)	
**Talkativeness**					
No	81 (39.3)	22 (44.9)	8 (34.8)	6 (42.9)	0.84 (0.841)
Yes	125 (60.7)	27 (55.1)	15 (65.2)	8 (57.1)	
**Social withdrawal**					
No	198 (96.1)	47 (95.9)	22 (95.6)	14 (100)	0.59 (0.899)
Yes	8 (3.9)	2 (4.1)	1 (4.4)	-	
**Suicidality**					
No	200 (97.1)	49 (100)	23 (100)	13 (100)	3.35 (0.341)
Yes	6 (2.9)	-	-	-	
**Grandiosity**					
No	186 (92.3)	45 (91.8)	22 (95.7)	14 (100)	2.19 (0.534)
Yes	20 (9.7)	4 (8.2)	1 (4.4)	-	
**Thoughtlessness**					
No	206 (100)	48 (98.0)	23 (100)	14 (100)	4.98 (0.174)
Yes	-	1 (2.0)	-	-	
**Poor appetite**					
No	191 (92.7)	45 (92.0)	22 (95.6)	12 (85.7)	1.30 (0.730)
Yes	15 (7.3)	4 (8.0)	1 (4.4)	2 (14.3)	
**Undressing in public**					
No	186 (90.3)	44 (89.8)	23 (100)	14 (100)	4.00 (0.264)
Yes	20 (9.7)	5 (10.2)	-	-	
**Irritability**					
No	191 (92.7)	45 (91.8)	20 (87.0)	10 (71.4)	**7.86 (0.049)**
Yes	15 (7.3)	4 (8.2)	3 (13.0)	4 (28.6)	
**Disorganized speech**					
No	196 (95.2)	21 (91.3)	21 (91.3)	14 (100)	1.53 (0.766)
Yes	10 (4.9)	2 (8.7)	2 (8.7)	-	
**Substance use**					
No	188 (91.3)	46 (93.9)	21 (91.3)	14 (100)	1.64 (0.651)
Yes	18 (8.7)	3 (6.1)	2 (8.7)	-	
**History of a stressor**					
No	160 (78.4)	32 (66.7)	15 (65.2)	8 (57.1)	6.45 (0.091)
Yes	44 (21.6)	16 (33.3)	8 (34.8)	6 (42.9)	
**Chlorpromazine**					
No	23 (11.2)	4 (8.3)	1 (4.4)	2 (14.3)	1.49 (0.685)
Yes	183 (88.8)	44 (91.7)	22 (95.6)	12 (85.7)	
**Haloperidol**					
No	165 (80.1)	41 (85.4)	20 (87.0)	10 (71.4)	2.09 (0.554)
Yes	41 (19.9)	7 (14.6)	3 (13.0)	4 (28.6)	
**Risperidone**					
No	2012(98.1)	48 (100)	22 (95.6)	14 (100)	2.09 (0.555)
Yes	4 (1.9)	-	1 (4.4)	-	
**Stela zine**					
No	205 (99.5)	47 (97.9)	23 (100)	14 (100)	1.75 (0.626)
Yes	1 (0.5)	1 (2.1)	-	-	
**Fluphenazine**					
No	187 (90.8)	39 (81.3)	21 (91.3)	12 (85.7)	3.88 (0.274)
Yes	19 (9.2)	9 (18.8)	2 (8.7)	2 (14.3)	
**Quetiapine**					
No	205 (99.5)	48 (100)	23 (100)	14 (100)	0.41 (0.937)
Yes	1 (0.5)	-	-	-	
**Mood stabilizers**					
**Carbamazepine**					
No	47(22.8)	11 (22.5)	1 (4.4)	2(14.3)	4.71 (0.194)
Yes	159 (77.2)	38 (77.6)	22 (95.6)	12 (85.7)	
**Sodium valproate**					
No	194 (94.2)	44 (89.8)	21 (91.3)	14 (100)	2.12 (0.547)
Yes	12 (7.1)	5 (10.2)	2 (8.7)	-	
**Antidepressants**					
**Amitriptyline**					
No	194 (94.2)	47 (95.9)	22 (95.7)	13 (92.9)	0.36 (0.948)
Yes	12 (5.8)	2 (4.1)	1 (4.3)	1 (7.1)	
**Fluoxetine**					
No	205 (99.5)	48 (98.0)	23 (100)	13 (92.9)	6.48 (0.0915)
Yes	1 (0.5)	1 (2.0)	-	1 (7.14)	

*Statistically significant at p-value 0.05; **Statistically significant at p-value < 0.001

### Annual rates of re-admissions among study participants stratified by the number of episodes of re-admission

#### First readmission (R1)

**Fig. 1:**
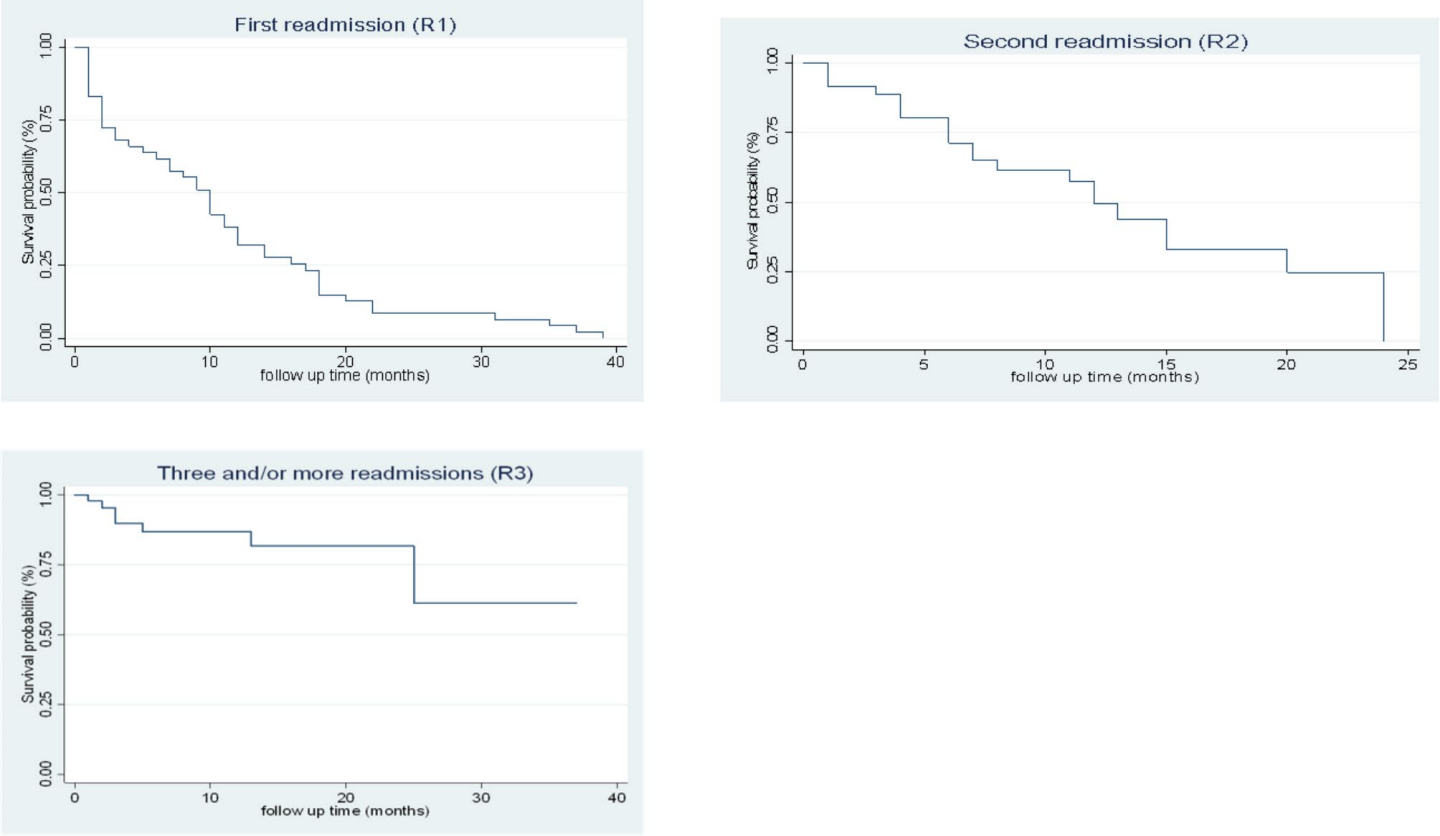
Kaplan-Meier survival function curves

The proportion of those who were readmitted in the first 12 months, for the first time following initial admission was 69.4% (*n* = 34/49), 22.5% (*n* = 11/49) from 13 to 24 months, 4.1% (*n* = 2/49) from 25 to 36 months, and 4.1% (*n* = 2/49) from 37 to 48 months (Table 3). The average duration to readmission was 10.7 months (*SD* = 9.8), *n* = 49. For the distribution of the data over time, see Fig. 1.

#### Second readmission (R2)

Following initial re-admission, the proportion of those who were readmitted was 78.3% (*n* = 18/23) in the first 12 months and 21.7% (*n* = 5/23) from13-24 months (Table 3). The average duration (in months) to the second readmission was 7.9 (*SD* = 6.4), *n* = 23.

#### Three or more readmissions (R3)

Following the second re-admission, the proportion of those who were readmitted in the first 12 months was 87.5% (*n* = 12/14) and 7.1% (*n* = 1/14) from 13 to 24 months (Table 3). The average duration to this readmission in months was 7 (*SD* = 6.6), *n* = 14.

**Table 3:** Readmission rate at different time intervals

Variable	Total number of readmissions	Mean time to readmission during study period. (in months) Mean (*SD*)	0 to12months *n* (%)	13 to 24months *n* (%)	25 to 36months *n* (%)	37 to 48months *n* (%)
R1	49	10.7 (9.8)	34 (69.4)	11 (22.5)	2 (4.1)	2 (4.1)
R2	23	7.9 (6.4)	18 (78.3)	5 (21.7)	-	-
R3	14	7 (6.6)	12 (87.5)	1 (7.1)	1 (7.1)	-

### Factors associated with readmission among study participants

The factors associated with the first readmission included the duration with symptoms before admission (in days), clinical presentations with symptoms of high energy levels, affective lability, poor appetite, and undressing in public. Symptom profile of poor appetite and undressing in public increase hazard ratio (HR) for readmission within any given time span while longer days with symptoms before admission, the clinical presentation with high energy levels, and mood lability were associated with reduction of the HR. For the second readmission, clinical presentation with food refusal increased the HR for readmission within a given timespan. (Table 4). No factors were associated with three or more readmissions.

**Table 4:** Factors associated with readmission

Variable	Hazard ratio (95% CI)	P value
*First readmission (Time from T0 to R1)*		
Duration with symptoms before admission (in days)	0.88 (0.79–0.97)	**0.011**
High energy levels	0. 23 (0.06–0.94)	**0.041**
Mood lability	0.001 (0.00–0.09)	**0.002**
Poor appetite	12.87 (1.36–121.44)	**0.026**
Undressing in public	19.65 (2.41–160.45)	**0.005**
*Second readmission (Time from R1 to R2)*		
Food refusal	150.88 (1.50–15206.55)	**0.033**

## Discussion

This study aimed at describing the rates and clinical factors associated with hospital readmission among individuals with BAD, admitted over four years (2018–2021) at a psychiatric unit in a tertiary hospital in Uganda. Our study findings are discussed in relation to findings in extant literature from other contexts to highlight important lessons on readmission and the associated clinical factors among BAD from a developing country context. Appropriate study implications for different groups of stakeholders involved in the care of BAD are highlighted below.

### Readmission among individuals with BAD

The re-admission rate was 23.8% during the entire study period, and approximately 69% of the readmitted participants were readmitted for the first time within the first 12 months of discharge. The readmission rate in the first 12 months in the present study is higher than 31.4% (*n* = 766) among 2443 individuals with BAD in a public hospital in USA in 2013 [34]. The lower readmission rate in USA may be due to the differences in sample size, and the psychiatric services provided, with Uganda struggling to provide adequate mental health services for its population [35]. For example, there is less use of second-generation or atypical antipsychotics in managing mood or psychotic symptoms and infrequently use of superior maintenance medications such as lithium in Uganda compared to the USA. In addition, the effect of well-developed community treatment teams and various rehabilitation services in the USA could also play a role in the lower re-admission rate in the first 12 months following discharge. Similarly, the rate of re-admission within 12 months in this study was also higher than 17.3% among patients with BAD in a study in Korea by Woo et al. (2014) [36]. The difference is likely because the study by Woo et al. (2014) included only individuals with first-episode bipolar mania receiving lithium or valproate and adjunctive atypical antipsychotics. Notably, the rates of readmission increased with the number of subsequent readmissions in the present study (i.e., the second and third readmission rates within 12 months were 78.3% and 87.5%, respectively). These findings are congruent with the finding in Switzerland by Kessing et al. (2004) that showed the rate of relapse with hospitalization to increase with the number of previous psychiatric admissions [37]. The plausible reason for this finding could be linked to the poor cognitive performance from constant neuroprogression associated with increasing number of bipolar episodes and causing long-term neuropsychological impact and worsening illness course and severity in affected individuals [38–40]. Implicitly as the course of BAD illness worsens, affected individuals are more likely to have frequent admissions as they experience more severe symptoms that may be more difficult to control.

### Factors associated with readmissions

The hazard ratio for readmission among individuals with BAD admitted in 2018 increased when individuals presented with poor appetite and/or were undressing in public. Both clinical symptoms are common symptom presentations of BAD in Uganda and may be associated with psychotic features [41, 42] or depression [43]. Poor appetite and food refusal in BAD is explained by higher levels of circulating leptin hormone [44] which inhibits the feeling of hunger. Leptin further directly inhibit serotonin production [45], yet low serotonin levels are linked with neuronal inflammation [46, 47]; subsequently resulting in neurodegeneration and worsening of the condition with resultant increased likelihood of readmission. Contrary to the finding in the present study, Perlick et al. (1999) highlighted that characteristic depressive symptoms, such as, poor appetite did not significantly alter the risk of readmission [19]. The reason for this discrepancy could be the difference in the time frame adopted for follow-up assessments of readmissions (i.e., 15-months vs. 4-years in our study). Additionally, active symptoms of depression often last longer, require frequent follow-up evaluation with psychotherapy and social support [43], which are aspects of mental health services that are not well established in the care model in Uganda [35]. However, Ford et al. (2015) reported that depressive symptoms were associated with the extreme valuing of happiness causing unnecessary disappointment each time one was not happy, a characteristic that was associated with readmission [48]. Additionally, depressive episodes last longer, and are more difficult to treat than the manic ones, fostering readmissions [43].

The present study also found that presenting with undressing in public increased the hazard ratio for readmission. Undressing in public among individuals with BAD could be indicative of increased sexual desire or psychosis [49, 50]. Increased sexual desire, a common presentation of the illness that is often worrisome [51], potentially predispose patients to sexual abuse, and confers an independent risk for readmission among individuals with bipolar disorder [52]. Increased sexual desire also results in increased incidence of risky sexual behavior, due to disinhibition of mania, which can result in contraction of sexually transmitted infections (e.g., HIV/AIDS) that can potentially worsen the course of the illness as a result of experiencing stigma from multiple illnesses and from neuro-complications of HIV [53]. Also, undressing cause public shame that leads to many patients feeling stigmatized or being stigmatized due to their mental health conditions, thus, stressing them and leading to readmission. Furthermore, presenting with undressing can be explained by the presentation being a disorganized behavior, hence psychotic in nature. Psychosis impairs cognitive and psychosocial functioning, indicating neuroprogression and worse illness severity [54].

High energy levels and mood lability were found to be protective against readmission. These findings are consistent with a follow up study by Perlick et al. (1999) which demonstrated that symptom profiles consistent with mania significantly decreased the risk for readmission [19]. Clinical symptom presentation with mood lability are protective, possibly because it is a dimension/symptom that occurs in personality disorders (e.g., borderline personality disorder) which is strongly linked to BAD [55], yet an individual is less likely to be hospitalized if their behavior has not changed from baseline and adjudged as features of a personality disorder rather than BAD. High energy levels were found to be protective against readmission possibly because they are associated with increased in socially acceptable activities in BAD [56], involving behaviors which may seem favorable in the society and compounded by the limited knowledge about mental illnesses in low income countries [57, 58], as lay people in the society may not be aware this is a mental illness symptom for presentation to hospital. Moreover, individuals with relapse BAD with high energy levels could pose a major challenge or difficulty for getting them to hospital. Hence, they could either be confined or shackled at home, or wander free as people are scared of them. Clinical presentation with irritability increased the risk for readmission in the present study following a recent discharge. This finding is likely because irritability is associated with worse illness severity as irritable patients are more likely to have limited social support and more functional impairment [59]. In support of this, disease severity is linked with higher relapse rates and admissions.

In contrast to previous studies our study found that increasing duration of days with symptoms before admission was protective against readmission. These discrepancies are likely due to poor help-seeking behavior previously reported in the study context, resulting in failure to return to hospital [60]. In addition, the poor help-seeking behavior is influenced by socio-cultural factors like myths and belief system about the causes of mental illness (e.g., mental illnesses are due to witchcraft, curses and evil or ancestral spirits). Accessibility, cost of care, stigma economic and health system-related factors can also be major barriers to early admission [61].

This study showed that the factors associated with the first readmission may not be the same as those associated with subsequent readmissions. Here are some possible reasons why: (i) in this study, the factors associated with each readmission were determined using data from the individual’s most recent hospitalization rather than their first hospitalization clinical characteristics (ii) BAD is a progressive illness, and the severity of the illness tends to increase over time. The first episode of hospitalization may be due to a relatively mild episode, whereas subsequent episodes may be more severe and may require different treatments [7, 62]. The differences in the severity of the episodes may also lead to differences in the factors that contribute to readmission [63]. (iii) The treatment of BAD can vary from person to person and may change over time with the treatment that was effective in preventing the first readmission may not be as effective in preventing subsequent readmissions [64]. Additionally, individuals may become non-adherent to their treatment regimen, which may contribute to readmission [65]. (iv) Psychosocial factors, such as stress, social support, and access to healthcare, that contribute to the first readmission may be different from those that contribute to subsequent readmissions [66]. For example, an individual may have a strong support system during their first hospitalization, but may lose that support over time, leading to differences in readmission risk.

In this study, more individuals received psychotherapy at each subsequent readmission. This could be because the mental health professionals were applying a preventative strategy to mitigate further readmissions since psychotherapy is effective in preventing BAD relapse, especially when combined with pharmacotherapy [67]. Administration of psychotherapy and other psychosocial care to readmitted patients can promote higher social functioning, and better coping strategies for BAD prodromes [68, 69]. Symptoms of uncoordinated speech and wandering away from home were less likely to be reported as the number of admissions increased. It is possible that those with these symptoms are more likely to be stigmatized and have a poor social support network due to the difficulty associated with communicating with the individuals with those symptoms [58]. Similarly, symptoms of aggression were less likely to be reported with subsequent admissions. Aggression is a predominant symptom of many psychiatric disorders and not pathognomonic of bipolar affective disorder [59]. However, aggressive behavior is common among individuals with BAD and is a major cause of hospitalization. This is contrary to the finding in our study most likely because the only studied domain was physical and not verbal aggression [59].

### Study implications

This study has important implications for individuals with BAD, caregivers, clinicians and other stakeholders. For individuals with BAD, the study provides insight into specific symptoms that increase the risk of readmission, such as those related to manic or depressive episodes. As shorter time to readmission results in more frequent readmissions, therefore information from this study can help patients and their caregivers to recognize early warning signs of relapse and take steps to prevent frequent readmission, such as being more vigilant about taking their medications and attending follow-up appointments. For caregivers, the study provides information on the specific needs of individuals with BAD, such as the importance of monitoring for signs of relapse and ensuring that prompt and adequate access to appropriate treatment.

To clinicians, the present findings provide insight into specific symptoms that increase the risk of readmission, and helps clinicians develop more effective treatment strategies to prevent readmissions. Additionally, by identifying clinical factors associated with readmission, this study encourages clinicians to develop more effective monitoring and follow-up programs for individuals with BAD, which can help to ensure appropriate care and support during treatment and recovery.

Furthermore, information obtained from this study can inform policy decisions related to healthcare access, funding, and coverage for individuals with BAD. For example, the study findings underscore the need for increased funding for community-based mental health services to manage clinical symptoms associated with readmission. Additionally, the study informs policy decisions related to early intervention and prevention, such as developing a better monitoring and follow-up program for BAD, which can help to ensure that patients receive appropriate care and support during treatment and recovery and help to reduce readmission rates.

This study also provides important insights for researchers in the field of mental health as the findings help to identify clinical factors, like specific symptoms or patterns of behavior associated with readmission. This information can be used to develop studies that will generate evidence for more targeted and effective interventions to increase interval to readmissions and improve overall outcomes for individuals with BAD. Overall, our study findings can play a crucial role in advancing the understanding of this mental health disorder, especially in developing countries context.

### Study limitations

This study’s findings should be interpreted with caution in view of the following limitations. First, this was a review of patients’ health management information system records, missing data are not uncommon in such study designs. To overcome this issue, we recommend that future researchers make provisions for multiple longitudinal assessments using prospective cohort studies. Second, the assessment of psychopathology was done without standardized instruments and depended on the ability of the clinicians to elicit these psychopathologies, hence it is possible that the estimates of psychopathology could be higher or lower than what was documented. We recommend the use of standardized instruments by future researchers to avoid estimation errors. Thirdly, many variables that could have influenced the findings, such as treatment adherence, personal circumstances, health system related factors and type of bipolar disorder, were not retrieved from the health management information system records of patients, and hence not included in the analysis. We recommend that future studies include these arrays of variables using prospective study designs to capture such information. The study did not capture the duration with symptoms for each reported symptom, thus the findings cannot determine which symptoms predict hospital readmission delay when intervention is not done promptly. Therefore, we recommend future researchers to have the duration of each symptom explored to identify this critical aspect of re-admission prediction. Finally, the data included in the analysis were from one facility, limiting the generalization of the findings. Although the sample of patients with BAD in our study cannot be representative of Uganda as a whole, it is reasonable to assume that the true estimate of readmission or relapse would be higher considering that a significant proportion of individuals with mental illnesses that do not receive orthodox clinical attention. Moreover, the sample size needed to be increased to identify potential associations between the variables of interest. However, even among those who receive orthodox clinical attention, only a fraction come to tertiary health facilities where the present study was conducted. Some patients may have presented to primary, secondary, or other private health facilities.

## Conclusion

In this study involving a retrospective review of cases of BAD spanning four years at a tertiary hospital in Uganda, it can be concluded that the rate of readmission of BAD is high and identifiable clinical characteristics were explanatory factors for time to re-admission. Also, the duration of clinical symptoms before admission was identified as a potentially modifiable factor for readmission. Follow-up of patients with BAD after hospital discharge is crucial as they have high readmission rates. Clinicians, case managers and social workers should pay more attention to clinical symptom at admission to inform assertive discharge planning, engagement of family members and community health education and rehabilitation to ensure continuity of care and reduce readmissions following hospital discharge. Associations with the first readmission for BAD may not be different for subsequent readmissions due to differences in disease progression, treatment, and psychosocial factors. Healthcare providers should take these factors into consideration when developing treatment plans for individuals with bipolar disorder to reduce the risk of readmission. However, further prospective studies looking at BAD are warranted to better understand extended causal relationships between predictors and readmission and inform optimal management strategies.

## Data Availability

The datasets will be made available to appropriate academic parties on request from the corresponding author.

## Abbreviations

HMIS: Health Management Information Systems
BAD: Bipolar Affective Disorder.
